# A Comparative Study of Interleukin 6, Inflammatory Markers, Ferritin, and Hematological Profile in Rheumatoid Arthritis Patients with Anemia of Chronic Disease and Iron Deficiency Anemia

**DOI:** 10.1155/2019/3457347

**Published:** 2019-04-01

**Authors:** Eman Tariq Ali, Azza Sajed Jabbar, Ali Nazar Mohammed

**Affiliations:** ^1^Department of Clinical Laboratory Sciences, College of Pharmacy, University of Basrah, Iraq; ^2^Department of Pharmacology, College of Pharmacy, University of Basrah, Iraq; ^3^Al Fayhaa General Hospital, Basrah, Iraq

## Abstract

**Background:**

Interleukin-6 (IL-6) proinflammatory cytokine is associated with the pathogenesis of rheumatoid arthritis and development of anemia in it. This is a comparative study of inflammatory and hematological parameters in RA patients with anemia of chronic disease (ACD) and iron deficiency anemia (IDA). It aimed to demonstrate the changes in serum level of IL-6, ferritin level, and hematological parameters in different groups of patients with RA and to find out the potential correlation between serum level of IL-6 and ferritin level and the relationship between serum level of IL-6 and iron status.

**Methods:**

The study included 89 patients from both sexes divided into four groups (group 1: 30 iron deficiency anemia (IDA), 59 RA; group 2: 20 RA-COMBI; group 3: 23 RA-ACD; and group 4: 16 nonanemic RA). These different groups were compared with a healthy group of 50 healthy individuals. Different blood parameters (WBC, RBC, HGB, HCT, MCV, and MCH) have been evaluated. Serum concentrations of IL-6, hsCRP, anti-CCP, and ferritin were measured in all patients and healthy individual using enzyme-linked immunosorbent assay ELISA.

**Results:**

There were significant changes in most of blood parameters between the groups, and there was a significant increase in the levels of IL-6 among RA patients. This increase was highly significant among RA-ACD patients in particular, and this elevation has been directly correlated with clinical indices of disease activity such as hsCRP, ESR, anti-CCP, and ferritin. There was an inverse relationship between ferritin and all iron status parameter, such as RBC, HGB, and haematocrit.

**Conclusion:**

IL-6 and ferritin level estimation may be workable tests to differentiate the patients with IDA and ACD in RA.

## 1. Introduction

Rheumatoid arthritis (RA) is a chronic progressive inflammatory disease of immune etiology that can affect many organs and systems in the body, mainly the joint synovial membrane. The pathology of the disease is characterized by formation of synovitis manifested by infiltration, many inflammatory cells such as lymphocytes and macrophages with evidence of hyperplasia and thickening of the synovial membrane with neovascularization and excessive secretion of synovial fluid. This results in the joints, swelling, stiffness, and arthralgia, ultimately leading to destruction of articular cartilage, bone erosion, and physical disability. Patients with RA may manifest various systemic symptoms such as fever, lethargy, fatigue, osteoporosis, muscles weakness, and anemia [1].

Anemia is a common characteristic disorder that affects the health of rheumatoid patients [2]. There are many types of anemia in RA such as iron deficiency anemia (IDA), anemia of chronic disease (ACD) or what is known as anemia of inflammation, the combination of both IDA and ACD, and hemolytic anemia [3]. It has been found that ACD is a common type of anemia in RA [4]. Although the pathogenesis of ACD is a complex process, many factors, particularly iron imbalance because of decrease in releasing of iron from mononuclear phagocytic system, absorption of iron, and decline in the ability of erythropoietin to respond to anemia, may contribute to the development of ACD [5].

Recent studies observed a possible role of interleukins and inflammatory cytokines as mediators in the pathogenesis of ACD [2]. IL-6 is a pleiotropic cytokine with different physiological activities including regulation of inflammatory processes, metabolism of bone, and immune response [5]. However overproduction of IL-6 may contribute to systemic inflammatory processes and induce cytokines production [6, 7].

Ferritin is a biochemical marker; its level increases in case of chronic inflammations. It reflects iron status in the body. In ACD patients serum ferritin values may be normal or increased because of retention of iron by reticuloendothelial system [8].

The current study is an attempt to examine the serum level of IL-6 and hematological profile in RA patients compared with healthy individuals and to detect the possible correlation between serum level of IL-6 and ferritin level and the relationship between serum level of IL-6 and iron status.

## 2. Materials and Methods

### 2.1. Study Design

The source of the data was the laboratory findings for patients who attended private authorized clinical laboratories. The study was conducted in Basra City, south of Iraq, between March 2017 and March 2018.

The study included 89 patients from both sexes defined as four groups. The first group was composed of 30 patients (23 females and 7 males; mean age 43 years) who fulfilled the criteria of iron deficiency (serum ferritin levels <20ng/ml), and these were classified as having IDA group. The other group was composed of 59 patients (of age 21-60 years) suffering from rheumatoid arthritis disease who fulfilled the revised 2010 American College of Rheumatology ACR/ELARCT criteria [9] and this group is further divided into 3 groups: the second group was made up of 23 RA anemic patients (16 females and 7 males; mean age 42.56 years) who had high/normal ferritin levels and these were categorized as having RA-ACD; the third group was composed of 20 RA anemic female patients (mean age 37.5 years) who had low/normal ferritin levels, and these made up the combination of IDA and RA-ACD (RA-COMBI); the fourth group was made up of 16 nonanemic RA patients (15 females and 1 male; mean age 33.6 years) who had normal ferritin levels. All RA patients included in the recent study suffered from moderate disease because the duration of disease was 3-5 years.

Demographic details and health status information such as gender, age at onset of disease, and duration of disease were recorded for each patient by questionnaire. Patients with known malignancies, renal failure, hemolytic conditions, chronic inflammatory diseases rather than RA and chronic blood loss, such as bleeding haemorrhoids, were excluded from the study. Fifty individuals (46 female, 4 male) of mean age 38.7 years (range 25-55 years) who looked apparently healthy and without any evidence of chronic inflammatory disease and anemia were considered as group 4.

### 2.2. ELISA Assay

#### 2.2.1. Estimation of IL-6 Cytokine

Serum concentrations of IL-6 were measured in all RA patients and healthy individuals using enzyme-linked immunosorbent assay (ELISA) according to the manufacturer's instructions (PeproTech, USA). The quantitative measurement of human ferritin concentration was estimated by ELISA according to the specifications provided by the manufacturer (POINTE scientific, Inc., USA) with regard to the corresponding concentration values in ng/ml. In IDA classification, a cutoff value of ferritin level, was taken as 10 ng/ml and 20 ng/ml in females and males, respectively, and any lower levels were considered as IDA.

#### 2.2.2. Estimation of Anti-CCP Level

Serum anti-CCP antibody was determined by ELISA using (IMMULISA-CCP assay kit, IMMCO-DIAGNOSTICS, USA), as described by the manufacturer. A standard curve was determined by plotting the optical density (OD) of each calibrator for the corresponding concentration values in U/ml. Samples ≥ 25 U/ml are defined as positive.

#### 2.2.3. Evaluation of hsCRP Level

hsCRP were evaluated in serum patients with RA and control groups by using ELISA according to the manufacturer's instructions (Demeditec, Germany). All the reagents supplied by this company were ready for use, such as MTP-International stander 5-vials, Chromogen Solution, Conjugate, and Stop solution, except Washing solution and specimen diluents. A standard optical density (OD) curve was created for each calibrator provided with the kit for the corresponding concentration values in mg/l. Samples > 3.0 mg/l are defined as high risk.

### 2.3. Hematology Profile

Different types of anemia were diagnosed by a specialist hematologist in a private laboratory. Two ml of venous blood in EDTA tubes has been used to measure different hematological parameters.

#### 2.3.1. Complete Blood Count (CBC) Test

Complete Blood Count (CBC) and White Blood Cell count tests were performed using the Auto Hematology Analyzer (Ruby, Germany). Patients with anemia were diagnosed according to the WHO criteria. Patients with hemoglobin value <12 g/dL in women and<13 g/dL in men were considered anemic.

#### 2.3.2. Erythrocytes Sedimentation Rate (ESR) Test

Erythrocytes Sedimentation Rate (ESR) was measured using Westergren method [10].

### 2.4. Statistical Analysis

Statistical analysis was carried out using Statistical Package for the Social Sciences (SPSS) software for Windows, version 24.0, IBM (SPSS Inc., IL, USA). The data are represented as a mean value ± standard deviation (SD). Comparison of group differences on normally distributed numerical variables was assessed by using the Independent Student's t-test (groups 1 and 2). One-way ANOVA (post hoc tests) was conducted for subgroup comparisons (groups IDA, RA-ACD, RA-COMBI, and healthy control) depending on the least significant difference (LSD) at a level less than 0.05 by using Gene State 2009. The Kruskal Wallis analysis was performed to analyze the results of ESR data. P-values at levels (p<0.05) were statistically significant. The Pearson correlation test was used to analyze the correlations between various laboratory findings and the significance level was measured by two-tailed paired test.

## 3. Results

### 3.1. Comparison between Healthy Control and Total RA Patient Groups

Table 1 shows the comparison between two main groups: 50-healthy-control group and 59-RA-patient group. The RA patients group included 51 (86.4%) females and 8 (13.6%) males. The mean age was 38.44±11.43 years.

Data analysis showed that there were significant differences in all hematological parameters between RA patients and control group (p<0.05) except in the MCH. RA patients had significant increases in WBC count (7.39±1.7 10^∧^3/*μ*L) (p=0.01) compared to healthy individuals (5.5±2.6 10^∧^3 /*μ*L), and there was a high significant increase in ESR for RA patients and healthy group (59.77 ± 4.34, 7.98 ± 3.76 mm/h), respectively (p≤ 0. 001).

Moreover, all immunological findings between total RA patients and control group were significantly different (p<0.05). Serum level of IL-6 of RA patients (483.32 ± 35.4 pg/mL) showed a high significant increase compared to that of healthy group (4.94±.0.349 pg/mL) (P= 0.0001).

### 3.2. Comparison of the Hematological Profile between Anemic RA and Nonanemic RA

The comparison showed significant differences in concentrations of RBC, MCV, and ESR (p=0.005, p= 0.006, and p=0.002), respectively. As seen in Table 1 the anemia, which is represented by the significant differences in hemoglobin concentrations and MCV, was more prevalent (72.8%) among RA patients (43 individuals) than nonanemic state (16 individuals). It was slightly more prevalent in female compared to male patients (83.70% vs. 16.30%). ESR was significantly increased in anemic RA (68.34±33.5mm/h) compared to nonanemic RA patients (36.75±19.6mm/h), p=0.002 (Table 1). All immunological findings between anemic RA and nonanemic RA were significant at level p< 0.05.

### 3.3. Comparison of Hematological and Immunological Parameters among Different Subgroups

Data analysis (Table 2) showed that there were significant changes between RA-ACD and RA-COMBI and the control group in WBC concentration (p≤0.001), while the difference between IDA and healthy control was not significant. RA-ACD patients showed the highest significant increase in WBC (8.67 ± 4.08 10^∧^3/*μ*L), p<0.05. Mean values of RBC concentrations were significantly decreased among the patients of the subgroups IDA, RA-ACD, and RA-COMBI, and the highest significant decrease was among the patients with RA-ACD (3.25 ± 0.60 10^∧^6/*μ*L) (P≤0.001).

HGB concentration, HCT, MCV, and MCH showed overall significant decreases among the patients of all subgroups studied compared to the control group. IDA patients had more significant decrease in these parameters (8.53±1.01 g/dl, 27.86±2.39%, 64.4±8.95fI, and 19.8±3.95pg), p<0.05.

The comparison between IDA and RA-ACD patients in hematological parameters showed significant differences in RBC and MCH (p<0.05), but nonsignificant differences in HGB, HCT, and MCHC.

As seen in Table 2 ESR values showed significant changes among the patients of the different subgroups and the highest significant value was among the patients with RA-ACD (72.56±30.5mm/h) (P≤0.001). In addition, patients with RA-COMB had higher significant change in ESR value (63.5±36.9 mm/h) (p≤0.001) than IDA patients and the healthy control group.

The comparison of immunological parameters among different subgroups and the healthy group showed that hsCRP level had significant changes. Its level was significantly higher in both RA-ACD and RA-COMBI patients (7.09 ± 2.3 and 7.18 ± 2.6 mg/L), respectively. The same result was reported about anti-CCP. There were higher values in the patients with RA-ACD and RA-COMBI subgroups (258.6 ± 43.5 and 324.3 ± 63.3U/ml), respectively (p≤0.001).

Serum levels of IL-6 also significantly varied among the subgroups and healthy group. As seen in the Table 2 RA-ACD patients showed a high significant increase (431.3 ± 52.2 pg/ml) (p≤0.001) compared to IDA patients (2.18 ± 1.4 pg/ml) and control group (4.94 ± 2.4pg/ml), while RA-COMBI patients showed a more significant increase (459 ± 61.8pg/ml) (p≤0.001). Also, significantly, ferritin levels showed the highest value in RA-ACD patients (83.39 ± 22.9 ng/ml) compared to IDA and RA-COMBI patients and the healthy control.

### 3.4. Correlation Assessment between IL-6 and Other Markers

Based on our assessment of whether the laboratory values are interrelated, a significant correlation was identified between the following parameters: IL-6 level was positively correlated to hsCRP (r=0.981, p= 0.0001), anti-CCP(r= 0.984, p≤0.001), ESR (r=0.468, p=0.03), and ferritin (r=0.534, p≤0.001) (Table 3).

### 3.5. Correlation Assessment between Ferritin and Iron Status

Data analysis revealed that there was a strong positive correlation between ferritin and ESR (r=0.726, P≤0.001), while it was an inverse correlation between both ferritin and RBC (r= 0.734, p≤0.001), HGB (r= 0.975, p = 0.0001), and HCT (r=0.475, p= 0.03) (Table 4).

## 4. Discussion

The hematological profile comparison between healthy control and RA patients group showed that there were highly significant differences in all hematological parameters such as RBC concentration, MCH, and MCHC. Hence the prevalence of anemia among RA patients was (72.8%) higher than the prevalence reported in RA patients from Western countries [11], which varies from 33.3 to 59.1%. The prevalence rate differs in different studies due to its association with the difference in definition of anemia [12]. This result is confirmed by previous report [13].

The anemia is a common hematological disorder among patients with RA. Many factors may explain this feature including iron deficiency, defect in the production of erythropoietin, decline in the response of bone marrow to erythropoietin, and a defect in the releasing of iron from reticulo endothelial system [13]. Furthermore, in study [14], a decline of iron as reflected by Hb and MCV was observed among patients with inflammatory diseases such as RA. This result is inconsistent with the result of the current study where HGB and MCV are significantly reduced among RA patients.

Moreover the prevalence of anemia was higher among female patients, 83.7%, than male patients, 16.3%. This finding is similar to that in study [13]. In general, there was a difference in sex ratio in suffering from RA; that is, female patients suffered from RA more than males. This difference in the RA ratio between males and females may be explained by the differences in the level of various sex hormones [15].

The study also showed a significant increase in WBC concentration compared to healthy control. This result is consistent with study [14], which found that RA patients suffer from leukocytosis due to an increase in immune activation [16].

The anemic RA patients showed significantly higher values of IL-6, hsCRP, and anti-CCP than those of nonanemic RA patients, which is due to the high activity of the disease, in particular in the RA-ACD patients. This finding was similar to that in a previous study [13]. Additionally the nonanemic RA patients showed significant differences from anemic RA ones in most hematological parameters: RBC, HGB, and MCV. This finding may be explained by the fact that nonanemic RA patients had better lifestyle than anemic RA patients [13].

The comparison between IDA and RA-ACD revealed significant differences in WBC, RBC, MCV, MCH, and ESR. In fact the pathogenesis of ACD is complicated and mainly because of changes in the balance of iron due to an increased immune activation [5]. A previous study reported that there are three immune mechanisms involved in the ACD development: a decrease in the RBC lifespan, impaired proliferation of erythroid cells, and elevated retention of iron in the RES. Therefore, patients with RA-ACD have an increased activity of the disease than patients with IDA [14], which is inconsistent with the result of the present study showing significant differences in the inflammatory markers such as ESR and CRP.

The present study showed that there were no significant differences in ESR between IDA and normal group, but ESR significantly increased among patients with RA-ACD compared to normal. The finding agrees with the finding of a previous study [13] which found that some RBC indices like MCH and MCHC have no significant differences between IDA and RA-ACD patients; hence they could not identify the patient of these two groups. But the red-blood cell indices such as MCV and MCH are conventionally considered important in identifying IDA patients and recognizing them. IDA patients showed significant decrease in the iron status, such as ferritin; the present study supposed that the low level of ferritin is one of the most critical brands that diagnose patients with iron deficiency and differentiate between IDA and RA-ACD patients.

The comparison of the immunological indicators showed significant differences between healthy and RA patients. It has been found that several inflammatory cytokines like IL-6 may be involved in the development of inflammatory diseases such as RA due to its ability to induce hypoferremia [17], and that is why ACD showed significant increase in the inflammatory indicators such as hsCRP and anti-CCP compared to the healthy control as mentioned above.

The difference is more confirmed between RA-ACD and a healthy group. The level of IL-6 is highly elevated in RA patients. The finding is harmonious with a study by [5] which found that the concentration of IL-6 was increased in the case of RA. An elevated level of IL-6 signaling may negatively affect homeostasis and IL-6 involved in the pathogenesis of immune diseases and chronic inflammatory conditions like RA [18] and play a main role in articular manifestation of the disease.

The RA and other chronic inflammatory diseases are directed by a complicated network of cytokines such as IL-6 that has the ability to perform many physiological functions such as proinflammatory response to infectious conditions [19–21]. This finding was also approved by another study [2] which found that the main mechanism in the pathogenesis of ACD in RA is due to increased IL-6, and it can inhibit erythropoiesis [22].

Anemia associated with chronic disease ACD is often normocytic, but the anemia can become microcytic and tends to be more severe in presence of concomitant IDA [23]. Some patients with RA chronic diseases such as RA-COMBI subgroup actually suffer from both iron deficiency and inflammatory anemia revealed more serious type of anemia.

Therefore, they showed more increase in concentrations of IL-6 compared to those in IDA and health controls. This finding suggests that proinflammatory cytokine Il-6 has been documented to be highly correlated with the severity of the disease. This result is confirmed by a previous study [24]. Another study confirmed that IL-6 production and developed anemia were linked by inhibition of iron metabolism and the formation of erythrocytes in the bone marrow [25]. This explanation was confirmed by a previous study which observed that the high production of Interleukin-6 at inflammatory sites stimulated the production of hepcidin, an acute phase protein, which might cause the iron to be isolated in reticuloendothelial as well as reduced absorption of intestinal iron [26]. Patients with RA-ACD had higher levels of inflammatory markers, such as increased leukocytes, hsCRP, ESR, and ferritin which were secreted under the influence of IL-6, than both IDA patients and the healthy controls. These results are consistent with the previous findings which demonstrated that IL-6, as the most important cytokine in inflammatory response, may be employed in the routine laboratory tests which are used in the diagnosis of ACD [27]. A recent study shows a significant increase in the level of ferritin in patients with RA-ACD and confirmed the existence of a positive correlation between IL-6 and level of each of hsCRP, anti-CCP, ESR, and ferritin. On the other hand, there was an inverse correlation between ferritin and each of RBC concentration, HGB, and HCT, which explains the high concentration of ferritin in patients with RA-ACD compared with those in the IDA, RA-COMBI, and healthy groups. This is compatible with the outcome of previous research [5] where its level increases with the severity of disease due to increased iron retention in the RES and activation of immunity.

## 5. Conclusions

One of the most common complications of rheumatoid arthritis is anemia of chronic disease. IL-6 is the main cause of developing anemia chronic inflammation and is expressed in excess at sites of inflammation.

IL-6 levels are considerably elevated in the serum of RA patients with ACD, and this elevation has been directly correlated with clinical indices of disease activity such as hsCRP ESR, anti-CCP, and ferritin. Therefore IL-6 and ferritin level estimation may be workable test to differentiate the patients with iron deficiency anemia and anemia of chronic disease in RA.

## Figures and Tables

**Table 1:** Demographic, hematological, and immunological parameters comparison between healthy control and RA patients and between anemic and nonanemic RA patients.

Comparison between	Healthy control and total RA patients			anemic RA and nonanemic RA patients		
Groups	Healthy control	Total RA patients	P≤	anemic RA	non- anemic RA	*∗*P<
Parameter	N=50	N=59		N=43	N=16	
Sex, F/M	46/ 4	51/ 8	NS	36/7(72.8)	15/1(27.2%)	0.001
Female%	92%	86.40%		83.70%	93.70%	
Male, %	8%	13.60%		16.30%	6.25%	
Age (years)	38.78±7.47	38.44±11.43	NS	40.23±10.74	33.6±12.2	0.02
	(25-55)	(21-60)		(24-60)	(21-58)	
WBC(10^∧^3/*μ*)L	5.5±2.6	7.39±1.7	0.01	6.62±0.412	5.20±0.619	NS
RBC(10^∧^6/*μ*L)	4.88±0.46	3.99±0.91	0.001	3.83±1.01	4.427±0.330	0.005
HGB (g/dl)	13.2±1.28	10.27±1.8	0.01	9.45±1.30	12.46±0.98	0.005
MCV(fl)	87.64± 5.7	82.8±7.64	0.05	81.62±8.38	86.06±3.69	0.006
MCH (pg)	27.12±2.43	26.83±4.09	NS	26.25±4.5	28.39±1.4	NS
MCHC(g/L)	31.87±1.17	32.01±3.52	0.02	31.65±4.01	32.99±1.30	NS
ESR (mm/h)	7.98±3.76	59.77±4.34	0.001	68.34±33.5	36.75±19.6	0.002
hsCRP (mg/L)	0.96±0.80	7.480±2.5	0.001	7.1±2.4	8.4 ± 2.5	0.03
Anti-CCP (U/ml)	2.4±1.46	325.5±34.7	0.001	289.2±37.4	423.1±75.9	0.01
IL-6 (pg/mL)	4.94±.342	483.32±35.4	0.0001	444.2±39.6	588.39±71.5	0.02
ferritin (ng/mL)	56.5 ±5.47	83.3±3.24	0.001	97.93±21.5	33.6±5.07	0.01

*∗*P is statistically significant at level < 0.05.

Data are reported as mean ± standard deviation (range).

*WBC*: white blood cells; *RBC:* red blood cells; *HGB*: hemoglobin; *HCT*: haematocrit; *MCV*: mean corpuscular volume, *MCH*: mean corpuscular hemoglobin; *MCHC*: mean corpuscular hemoglobin concentration; *ESR*: erythrocyte sedimentation rate; *hsCRP*: high sensitivity C-reactive protein, *IL-6*: interleukin 6, *anti-CCP*: anti-cyclic citrullinated peptide; *NS*: not significant; F: female; M: male.

**Table 2:** Comparative analysis of immunological and hematological marker indices in patients with IDA, RA-ACD, and RA-COMBI and healthy control group.

Parameter	IDA	RA-ACD	RA-COMBI	Control	*∗*P≤	1 vs 2	1 vs 3	1 vs 4	3 vs 4	3 vs 2	*∗∗*2 vs 4
No. patients	N=30	N=23	N=20	N=50							
Sex, F/M	23/7	17/6	20/0	46/4	-	-	-	-	-	-	-
Age (years)	43±7.32	42.56±11.06	37.55±9.95	38.78±7.47	NS	NS	NS	NS	NS	NS	NS
Rang	(25-60)	(24-60)	(24-55)	(25-55)							
WBC(10^∧^3/*μ*)	6.36±2.70	8.67±4.08	4.76±2.50	5.5±2.6	0.001	0.003	0.001	NS	0.001	0.001	0.001
RBC(10^∧^6/*μ*L)	4.4±0.69	3.25±0.60	4.49±0.99	4.88±0.4	0.001	0.001	NS	0.002	NS	0.001	0.001
HGB (g/dl)	8.53±1.01	9.15±1.37	9.79±1.16	13.21±1.28	0.001	NS	0.001	0.001	0.001	NS	0.001
HCT (%)	27.86±2.39	28.46±4.96	31.54±9.51	41.41±3.83	0.001	NS	0.01	0.001	0.001	NS	0.001
MCV(fl)	64.4±8.95	85±6.24	74.7±4.8	87.64± 5.7	0.001	0.001	0.001	0.001	0.001	0.001	0.001
MCH (pg)	19.8±3.95	28.6±4.76	23.5±2.37	27.12±2.34	0.001	0.001	0.001	0.001	0.001	0.001	0.001
MCHC gl/dl	30.61±2.58	31.77±5.26	31.51±1.84	31.87±1.17	NS	NS	NS	NS	NS	NS	NS
ESR (mm/h)	6.1±1.2	72.56±30.5	63.5±36.9	7.98±3.7	0.001	0.008	NS	0.001	0.001	NS	0.001
hsCRP (mg/L)	1.26±0.96	7.09± 2.3	7.18±2.6	0.96±0.11	0.001	0.001	0.001	NS	0.001	NS	0.001
Anti-CCP (U/ml)	2.41±1.32	258.6±43.5	324.3±63.3	2.4±1.4	0.001	0.001	0.001	NS	0.001	NS	0.001
IL-6 (pg/ml)	2.18±1.4	431.3±52.2	459±61.8	4.94± 2.4	0.001	0.001	0.001	NS	0.001	NS	0.001
ferritin(ng/ml)	7.17±1.00	83.39±22.9	8.38±1.31	56.5±30.7	0.0001	0.001	NS	0.001	0.001	0.001	0.001

**∗** *P* is statistically significant among the groups at level*< 0.05*. Data are reported as mean ± standard deviation (range).

*∗∗*Multiple comparisons among 4 groups depended on LSD.

*IDA*: iron deficiency anemia; *RA-ACD*: anemia of chronic disease; *RA-COMBI*: combination of IDA and ACD. F: female; M: male.

**Table 3:** Correlation between IL-6 and other inflammatory markers in RA patients.

Variables		hsCRP	anti-CCP	ESR	Ferritin
IL-6	r	0.981*∗∗*	0.984*∗∗*	0.468	0.534
	P	0.0001	0.001	0.03	0.001

*∗∗* Correlation is significant at the 0.01 level (2-tailed).

*∗* Correlation is significant at the 0.05 level (2-tailed).

**Table 4:** Correlation between serum ferritin and iron statuses in RA patients.

Variables		ESR	RBC	HGB	HCT
Ferritin	r	0.726*∗∗*	-0.734*∗∗*	-0.975*∗∗*	-0.475*∗*
	P	0.001	0.001	0.0001	0.03

*∗∗* Correlation is significant at the 0.01 level (2-tailed).

*∗* Correlation is significant at the 0.05 level (2-tailed).

## Data Availability

The data used to support the findings of this study are included within the article.
